# Dentistry for the Masses

**Published:** 1919-10

**Authors:** 


### DENTISTRY FOR THE MASSES

Social movements which are justified by public necessity usually develop by a series of impulses which are cumulative in effect, and they often establish themselves as the result of some final crisis which compels recognition of their importance.

A generation ago, W. D. Miller published his investigations of the human mouth as a focus of infection, by which he scientifically established two fundamental facts: First, that the human mouth is the habitat and breeding-ground of a large group of micro-organisms, a great proportion of which are pathogenic; and second, that organisms originating in the oral cavity may be carried to other parts of the body and thus set up disease in organs and tissues remote from the oral cavity. He further showed that septicemia may result from the absorption of toxins produced by bacterial activity in the mouth.

These pioneer researches, notwithstanding their far-reaching significance, were practically ignored, except as being interesting scientific observations, until Hunter in 1910 (see *Dental Cosmos*, July, 1918, page 585) presented the same problem from the standpoint of his clinical studies, and drew attention to the havoc that was being wrought to human health and efficiency by diseased teeth, and more particularly by ignorantly conceived and badly constructed dental prosthetic restorations. Both of these classic communications gave force and substance to the so-called Oral Hygiene movement as a practical application to the preservation of the public health and prevention of disease, based on these known facts regarding mouth infection.

Statistics soon became available which demonstrated beyond reasonable doubt that a sound body is in large degree dependent upon wholesome mouth conditions; and, concomitantly, the far-reaching importance of the relation of specific mouth disorders to obscure lesions of the vital organs, the special sense organs, diseases of the nervous system, etc., has been gradually unfolded by various investigations, until to-day the belief in the dental origin of disease is flourishing with an exuberance that is suggestive of hysteria. And then came the world war, which has added what ought to be the final demonstration, if any were needed, that proves the necessity for a healthy mouth and teeth as an insurance against bodily disease.

Military medical records of all nations furnish convincing evidence of the direct relation between sound teeth and the physical efficiency of the fighting men. Our government practically recognized this in the creation and equipment of the largest and most efficient army and navy dental service of any of the belligerents, and further emphasized its recognition of the vital importance of oral hygiene by providing for the instruction of its military forces in the personal hygienic care of the mouth.

These several influences, which have been operating more or less actively during the past generation, have now reached a critical stage of development. The cumulative effect of the several activities here referred to has been to place dental service in an entirely new position; that is to say, it can no longer be properly regarded as a luxury to be enjoyed exclusively by the rich and well-to-do—for, as a matter of fact the verity of which is supported by incontestable evidence, it is a vital necessity for the maintenance of public health. Because of existing conditions, because a physically sound citizenry is essential to the national welfare, because good health is fundamental in its relation to our national progress, our national well-being, and our national defense, and because sound teeth in a healthy mouth are essential to the physical welfare of our people, the dogma that dentistry is a luxury is indicative of a policy and mental attitude both limited in scope and selfish in conception, for which reasons alone it is untenable. The benefits of dental service must be made accessible to all the people in the same measure that the benefits of all phases of the healing art have been made generally accessible.

It is undoubtedly true that a certain type of dental service is a luxury, and that even that type must and doubtless does embody the highest ideals of health-conservation and disease-prevention; but it is also coupled with a luxurious ideal upon the part of those who demand, as well as those who render that type of service; and it is just that element—the demand for the luxurious and the acquisitiveness of those who cater to it—that obscures the problem of bringing a health-promoting dental service within the reach of the rank and file of humanity.

It is an economic as well as a humanitarian problem. The cost of bringing good, serviceable dental care to children of school age, the expense of whose education is borne by the taxpayer, is shown by reliable statistics to be somewhere around one dollar per annum per capita. The amount varies more or less according to circumstances. The cost of doing this humanitarian work is insignificant as compared with the price which we are now indirectly paying for not doing it, in doctors' bills, in the price we pay for re-education of mentally retarded children, in supporting institutions for defectives, and above all in the loss of physical efficiency in the man power of the nation which oral infection entails. Collectively, the dental profession, to its credit be it said, has promptly responded in full measure to every reasonable demand that has been made upon it. When the light of truth has made the path clear the dental profession has followed it. Everything now points to the democratization of dental service. In England it has been made a national question as a feature of the public health service.

The problem involves many details, among which are:

A greatly increased number of dentists.

Legalization, in all of the states, of the work of the dental hygienist, as it is now legalized in fifteen states.

Reorganization of the dental educational curriculum in recognition of the fact that educational provision should be made for two classes of dentists—(a) those who are to intelligently and adequately serve the public, and (b) those who are being educated as teachers and researchers.

The enactment of the state legislation which will make dental service an integral part of the public health activities of each state, under the administration of dentally educated executive officers.

The introduction into the public school curricula of obligatory education in oral hygiene.

The systematic oral inspection of school children and treatment of their dental defects by dentists.

The convincing argument in favor of such a departure is the economic one that it will pay, and pay well, to do it; for the cost is relatively small and the returns large. That the cost is small becomes evident when we rid our minds of the obsession that "Dentistry is a luxury." Luxurious dentistry is undoubtedly costly, but dentistry for the attainment of oral hygiene is not necessarily so.

In 1910, the late Dr. Percy B. McCullough set forth in a communication to the Academy of Stomatology the details of a system of dental service which he had put into successful practice at the dental dispensary of the department of Health and Charities in Philadelphia. In principle this plan had for its objective the securing of hygienic mouth conditions for the greatest number of school children at the least cost to the taxpayer, and the results obtained demonstrated the soundness of the plan. In detail it would be modified to-day, but in principle, and so far as its ultimate objectives are concerned, it was a successful pioneer effort in the direction here under consideration, and which is now in an acute stage awaiting practical solution. The world is now grappling with the problems of reconstruction. One of the greatest of these is the demand of the masses for improvement in living conditions, and all measures for the promotion of better general health are being actively pushed forward for consideration.

The problem of the dental profession in the activities of reconstruction is and will continue to be the carrying of dental service to the masses as a public health measure. It is fully time to set about it.—Editorial in September *Cosmos.*

